# Assessing congestion using estimated plasma volume status: Ready for prime time?

**DOI:** 10.1002/ehf2.15025

**Published:** 2024-08-12

**Authors:** Phuuwadith Wattanachayakul, Veraprapas Kittipibul, Marat Fudim

**Affiliations:** ^1^ Department of Medicine Jefferson Einstein Hospital Philadelphia Pennsylvania USA; ^2^ Sidney Kimmel Medical College Thomas Jefferson University Philadelphia Pennsylvania USA; ^3^ Division of Cardiology, Department of Internal Medicine Duke University School of Medicine Durham North Carolina USA; ^4^ Duke Clinical Research Institute Durham North Carolina USA

Identifying congestion is challenging, as up to 40% of patients deemed ‘dry’ on cardiovascular physical examination still have residual congestion. Characterized by unrecognized fluid retention or elevated filling pressures, this residual subclinical congestion frequently leads to early heart failure hospitalization (HFH) if managed with suboptimal decongestive strategies.^1^ Therefore, there is a need for a reliable tool for assessing congestion that is simple, non‐invasive and easy to implement. Estimated plasma volume status (ePVS) has been investigated as a surrogate for clinical intravascular congestion. It can be simply calculated using the Strauss‐derived Duarte formula: ePVS = 100 × (1 − haematocrit)/haemoglobin.^2^ Congestion, as indicated by increased ePVS, is linked to adverse cardiovascular outcomes in patients with heart failure with reduced ejection fraction (HFrEF) following acute myocardial infarction (AMI).^3^ However, whether ePVS at the time of discharge can predict long‐term outcomes in AMI patients remains unknown.

In this issue of *ESC Heart Failure*, Nogi *et al*. explore the association between intravascular congestion, measured by ePVS using the Strauss‐derived Duarte formula, at the time of discharge and long‐term clinical outcomes, including all‐cause deaths, cardiovascular deaths and HFH in patients hospitalized for AMI.^4^ The study included a large cohort of 1012 patients, 82.5% of whom were hospitalized for ST‐elevation myocardial infarction (STEMI). Using the established ePVS cut‐off of 5.5%, 36% of patients were included in the high‐ePVS group. Over a median follow‐up of 50 months, patients in the high‐ePVS group had a significantly higher incidence of all‐cause and cardiovascular deaths as well as HFH. After adjusting for established prognostic factors for AMI, high‐ePVS status remained significantly associated with a 1.9‐fold increase in all‐cause deaths. Further analysis demonstrated the association between ePVS status and echocardiographic parameters at discharge and at 1 year follow‐up. Patients in the high‐ePVS group had a higher degree of structural abnormalities [i.e., a higher left ventricular (LV) mass index and a lower LV ejection fraction (LVEF)]. Multiple linear regression analysis showed a significantly greater increase in LVEF and decrease in LV volume index over time in the low‐ePVS group.

The proper interpretation of the study relies on the premise that ePVS is a good surrogate for intravascular congestion. A key question remains: How well does ePVS assess intravascular volume compared with direct blood volume (BV) measurement? The current gold standard for BV measurement employs the indicator‐dilution technique, which involves the intravenous injection of a low‐dose radioisotope tracer, followed by the analysis of serial blood samples.^5^ This technique allows precise and reproducible measurement of BV, plasma volume and red blood cell volume. A study in patients with stable chronic HF found a weak correlation between ePVS using the Strauss‐derived Duarte formula and the directly measured plasma volume (*r* = 0.29).^6^ Furthermore, changes in ePVS similarly had a poor correlation with changes in direct BV measurement (*r* = 0.16). The study also explored the ePVS using the Kaplan–Hakim formula, which also accounts for sex and lean body weight. Despite that, only a moderate correlation with directly measured plasma volume was observed (*r* = 0.64). Furthermore, even under the most optimistic assumption that ePVS accurately represents intravascular congestion, the prognostic implications of intravascular congestion are conflicting, even when measured directly using the gold standard technique. For example, a single‐centre study of both patients with chronic and recent worsening HF found an association between large BV expansion and a higher HFH risk in patients with chronic HF but a lower HFH risk among those with worsening HF.^7^ In contrast, a multi‐centre study of chronic HF patients found no significant difference in the HFH rate in patients with mild‐moderate or large BV expansion compared with those with low‐normal BV status.^8^ This reflects the complexity and variability of the impact of intravascular congestion across the HF disease spectrum.

Although ePVS is primarily intended to reflect intravascular congestion, its predictive value for adverse outcomes in this study may be influenced by additional factors beyond congestion itself. Considering the baseline characteristics of the study population, patients in the high‐ePVS group were unquestionably sicker, with more severe disease presentation, higher comorbidity burdens and lower haemoglobin levels. Consequently, the higher rates of all‐cause mortality, cardiovascular mortality and HFH observed in these patients could be attributable to known and unknown clinical factors beyond residual congestion, even after meticulously executed statistical adjustments. Given the dynamic nature of congestion, it is challenging to ascertain that the adverse impact of high ePVS on long‐term outcomes is simply from intravascular congestion, especially without repeated ePVS measurements. Moreover, using only haemoglobin and haematocrit levels in the ePVS calculation might oversimplify congestion assessment. Anaemia, by itself, is a well‐established poor prognostic predictor in patients with AMI and HF.^9^ As anaemia increases ePVS, it is difficult to discern if the poor prognosis associated with elevated ePVS stems from anaemia, intravascular congestion or both. While ePVS may be effective for the general population without extreme anaemia, it is less reliable in patients with AMI, where anaemia from anticoagulation‐related bleeding and catheterization complications are common, potentially affecting the ePVS and its interpretation. Perhaps ePVS might be more suitable in an outpatient setting after the patient has recovered from the acute phase, minimizing factors that could skew its interpretation of actual intravascular congestion.

More severe structural abnormalities at discharge and limited improvement in LV mass index and LVEF over 1 year in the high‐ePVS group suggest that high ePVS at discharge is a poor prognostic marker for LV remodelling. However, the mechanisms underlying the detrimental effects of high ePVS on LV remodelling need further exploration. It might be partly true that high ePVS, indicating residual intravascular congestion, leads to more neurohormonal activation and subsequent adverse LV remodelling. As high ePVS also represents a sicker population, a similar assumption can be made that the higher degree of structural abnormalities in the high‐ePVS group is influenced by other clinical factors that can also lead to neurohormonal activation and LV remodelling.^10^ Missing echocardiographic data in 15% of patients at 1 year has complicated the interpretation of the results. Hypothetically, more deaths in the high‐ePVS group could result in relatively less severe LV structural abnormalities among those who survived. This could lead to an underestimation of the true difference in LV improvement between the groups. However, the study did not clarify the extent of missing data attributable to deaths, nor did it address whether this missing data was balanced between the high‐ePVS and low‐ePVS groups.

While the accuracy of ePVS in reflecting actual intravascular status is uncertain, this study highlights the prognostic significance of ePVS in AMI that warrants further investigation to understand its practical implications, as shown in Figure 1. First, additional research is needed to determine whether ePVS can provide prognostic value beyond that of well‐established congestion markers such as clinical examinations or N‐terminal pro‐brain natriuretic peptide (NT‐proBNP).^11^ Second, identifying the optimal ePVS cut‐off specifically for AMI is crucial, as the cut‐off of 5.5% employed in this study was derived from the HF population, and congestion develops differently in AMI and HF.^12^ HF patients are accustomed to intravascular congestion and might tolerate higher ePVS. On the other hand, AMI patients develop congestion relatively acutely through myocardial ischaemia, causing impaired LV relaxation and reduced LV compliance.^13^ This translates to a lower tolerance for intravascular congestion in AMI, which might require a lower ePVS cut‐off for prompt detection and management. Lastly, although ePVS can be easily obtained and followed longitudinally,^3^ the effective strategy for managing patients with high ePVS is unknown. This is clinically significant because even NT‐proBNP, a well‐known prognostic congestion biomarker, has not shown a mortality benefit when used to guide therapy in HFrEF patients.^14^

**Figure 1:**
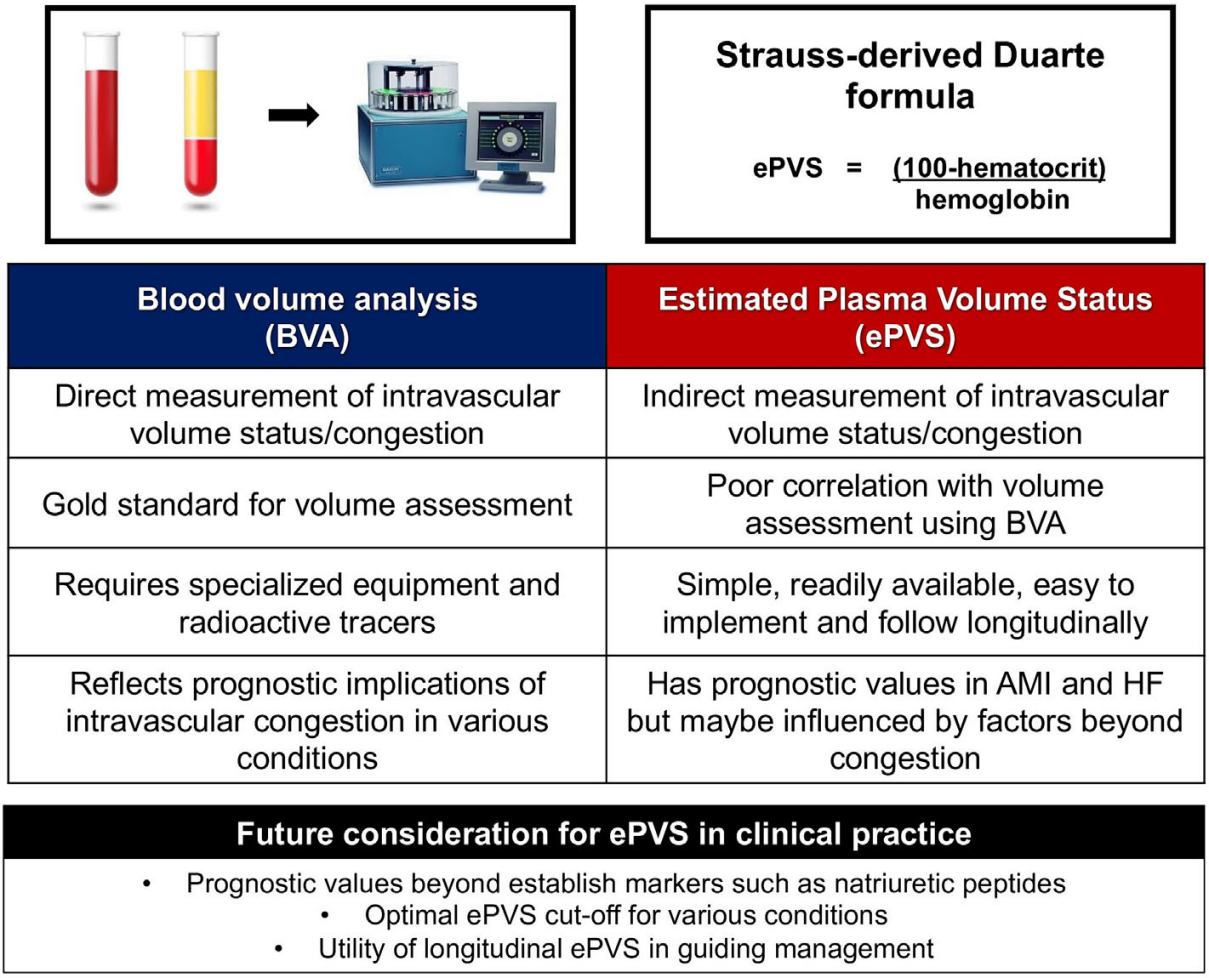
Assessing intravascular congestion: BVA versus ePVS and future considerations. AMI, acute myocardial infarction; BVA, blood volume analysis; ePVS, estimated plasma volume status; HF, heart failure.

ePVS shows promise as a simple prognostic tool, but this is just the first step. To integrate ePVS into clinical practice effectively, it is crucial to understand what it truly represents and establish clear clinical actions. Identifying scenarios where ePVS outperforms or complements existing congestion biomarkers is essential. Verifying ePVS efficacy through correlation with other remote monitoring HF devices could enhance its utility. Addressing these aspects robustly will be essential before ePVS can become a reliable and actionable biomarker in routine clinical care.

## Funding

The project received no financial support.

## Conflict of interest statement

Dr Fudim is supported by the NIH (1OT2HL156812‐01; 1R01HL171305‐01) and Doris Duke. He received consulting fees from Abbott, Ajax, Alio Health, Alleviant, Artha, Audicor, AxonTherapies, Bayer, Bodyguide, Bodyport, Boston Scientific, Broadview, Cadence, Cardioflow, Cardionomics, Coridea, CVRx, Daxor, Deerfield Catalyst, Edwards LifeSciences, Echosens, EKO, Feldschuh Foundation, Fire1, FutureCardia, Galvani, Gradient, Hatteras, HemodynamiQ, Impulse Dynamics, Intershunt, Medtronic, Merck, NIMedical, NovoNordisk, NucleusRx, NXT Biomedical, Orchestra, Pharmacosmos, PreHealth, Presidio, Procyreon, ReCor, Rockley, SCPharma, Shifamed, Splendo, Summacor, SyMap, Verily, Vironix, Viscardia and Zoll. All other authors have no relevant financial disclosures.
